# Intelligent Animal Husbandry: Present and Future

**DOI:** 10.3390/ani14111645

**Published:** 2024-05-31

**Authors:** Elena Kistanova, Stanimir Yotov, Darina Zaimova

**Affiliations:** 1 Institute of Biology and Immunology of Reproduction, Bulgarian Academy of Sciences, 1113 Sofia, Bulgaria; 2 Department of Obstetrics, Reproduction and Reproductive Disorders, Trakia University, 6000 Stara Zagora, Bulgaria; stanrad@abv.bg; 3 Department of Industrial Business and Entrepreneurship, Faculty of Economics, Trakia University, 6000 Stara Zagora, Bulgaria; dzaimova@gmail.com

The main priorities in the contemporary breeding of different animal species have been directed toward the use of intelligent approaches for accelerating genetic progress, ensuring animal welfare and environmental protection by reducing the release of manure and gas emissions [1,2,3,4]. An innovative way to achieve accelerated genetic progress in cattle breeding is the use of sexed semen from bulls with high genetic potential, the development of effective estrus synchronization protocols, and the application of innovative systems for artificial insemination. This, in turn, leads to the production of animals with very good productive traits and can reduce herd sizes [5,6]. Animal welfare requires strict control of the environment on the farm level by using automated sensor systems and up-to date diagnostic methods for the early detection of possible diseases. These can detect different changes in the environment, animal behavior, and some indicators characterizing their health status [1,7,8]. Housing a smaller number of animals in a good health condition, but with better productive qualities, is associated with a higher profitability of the farm and the production of lower manure masses, which has a crucial role in protecting the environment and reducing gasses from the animal husbandry industry [4,9]. Digitization of the technological process and the implementation of computer algorithms for processing the received information enable the adequate analysis of all factors influencing the production of milk and/or meat with high quality and making correct management decisions [10,11].

This Special Issue presents experimental and review material for discussing a wide spectrum of questions related to “Intelligent Animal Husbandry”, from the use of modern technologies for genetic progress in animals, to the application of information and communications technologies (ICT) to control the behavior and breeding conditions of animals, to intelligent waste management in animal husbandry (Figure 1). The publications included in the collection reflect an opportunity for intelligent approaches and smart solutions for raising different species of farm animals: dairy animals, such as cows and buffaloes [6,12]; pigs [4,13]; silkworms [14]; rabbits [8]; and chickens [11].

A major challenge in animal husbandry is the rearing of certain sexes of animals for specific purposes. This facilitates acceleration of the genetic progress in the herd, saving resources and reducing waste [2]. Sexed semen gives the ability to produce animals of a precisely defined and desired sex. However, semen sexing technology is still very expensive. The research by Yotov et al. published in this SI [6] contributes to solving this problem through economic and reproductive success by using sexed sperm. The authors proved that the application of a number of intelligent approaches as the presynchronization treatment of cows with the Ovsynch+PRID+eCG protocol, and the preliminary selection of candidates for insemination through ultrasound examination of their ovarian status, for example, can ensure higher efficiency of the artificial insemination of dairy cows with sexed sperm [6]. Accurate identification of the species and sex of pupae is also essential for silkworm sericulture, particularly for silk quality. The paper by He et al. [14] offers an economical and intelligent solution for sericulture breeding based on validation of the effectiveness of machine learning and deep learning in recognizing the species and sexes of pupae through image analysis. Additionally, this research has a future perspective in the development of a finer-grained neural network for improving the detection of subtle variations across species and sexes using datasets from diverse breeding batches [14].

The other problem of animal husbandry, requiring intelligent approaches for recognition and prediction, is animal behavior. The article by Stepancheva et al. [12] presents new data concerning the estimation of milking temperament scoring and its relationship with productive traits in cows of the buffalo breed Bulgarian Murrah. Machine milking includes physical and psychological stressors, which can negatively affect the milk yield in the dairy animals [1]. Stepancheva et al. [12] emphasize that buffalo behavior during milking is an extremely important tool for the determination of animals’ temperament and their suitability for farms with intensive management systems and the mechanization of daily production activities.

An interesting review paper by Ward et al. [13] elucidates the problems of pigs’ behavior on farms related to the tail biting (TB). Decision support tools (DSTs) are any interactive computer-based system intended to help identify, take a course of action, and solve problems of TB [15]. The results of this review outline the potential for a novel DST model that rely on objectively collated environmental-, animal-, and human-related data through real-time tracking technologies and can offer valuable assistance in managing and reducing TB risk on farms [13].

The application of new diagnostic tools is very important for the welfare and reproductive health of animals. The bulbourethral gland of rabbits has a crucial role in the production of semen with high quality and its transport to the female copulatory organ during ejaculation [16]. The investigation by Dimitrov et al. presented in this SI [8] supports the notion that magnetic resonance imaging (MRI) is a definitive and innovative method for studying this accessory gland in rabbit, allowing differentiation of the glands from neighboring soft tissue structures. The authors stress that MRI, an intelligent and non-invasive technique, could improve knowledge for the assessment of the reproductive system status in small farm animals.

Why does the raising of chickens require more environmental control than raising other meat-producing animals? Is fully autonomous microclimate control possible in poultry farms? The paper by Shivarov et al. [11] gives the answers to these questions. The authors developed and applied a cyber–physical system (CPS) for the intelligent management of a poultry farm for broiler meat production. The proposed CPS demonstrated high productivity with minimal production waste, at optimized costs and with a minimization of human errors [11].

The accumulation of waste of animal origin is one of the biggest problems in animal husbandry. Inadequate manure management is a major contributor of ammonia (NH_3_) and greenhouse gasses emissions that occur at all stages of manure production and handling [9]. The article by Benedek et al. [4] reflects a representative survey of pig farms to assess housing and manure management technologies in the Hungarian pig sector. During the survey, a new expert-based calculation method for converting farm data on pig numbers was developed. The survey methodology helped to elaborate an ammonia emissions calculator and knowledge transfer tool (AGEM-S) for use by farmers [4].

We understand that the present Special Issue does not cover all aspects of intelligent animal husbandry. Many debatable questions in different animal species remain, and represent the scope for further studies. Future investigations in intelligent animal husbandry could aim to implement advanced digital tools and artificial intelligence (AI) to process and analyze the vast amounts of data received from various sensor systems [17]. Nevertheless, certain unresolved issues still exist. Sometimes, information can be limited or difficult to access, which can limit the effectiveness of artificial intelligence [18,19]. Unlike the accurate monitoring of some physiological parameters, the ability of AI to understand the emotional states and behaviors of animals is very limited; this represents a potential risk of misclassifying their welfare. Additional problems may arise due to the need for the continuous learning of AI and the personnel who will use this new technology [20,21]. This suggests that future efforts to develop intelligent animal husbandry systems should be directed simultaneously in both directions—digitalization of the livestock sector with the introduction of innovative technologies, and training personnel to work with these tools.

We believe that the publications in this Special Issue, “Intelligent Animal Husbandry”, will provoke broad discussions around intelligent approaches in animal husbandry, as well as of their benefits and disadvantages. The authors acknowledge the Bulgarian National Scientific Program, “Intelligent Animal Husbandry” (MES-Bulgaria, D01-62/18 March2021) for their ideas, contributions, and support during the editing of this Special Issue. Additionally, we appreciate all these SI articles being published in the journal “*Animals*”, exploring the problems of intelligent organization of animal husbandry, collected in one valuable handbook for farmers; this could facilitate management based on the latest achievements in artificial intelligence being successfully introduced in farm animal practice.

## Figures and Tables

**Figure 1:**
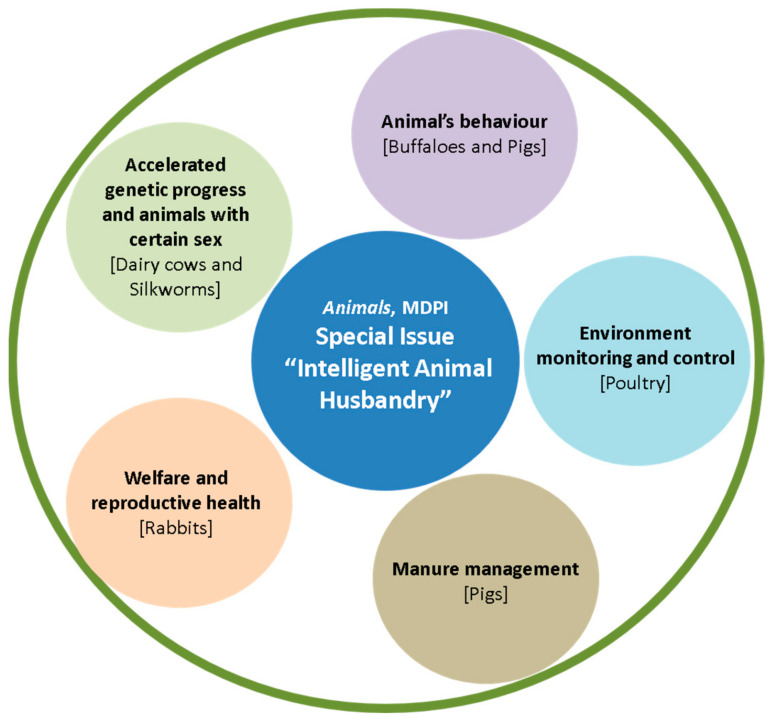
Topics and livestock species covered by the papers in this Special Issue “Intelligent Animal Husbandry”.

## References

[1] Jacobs J.A., Siegford J.M. (2012). Invited review: The impact of automatic milking systems on dairy cow management, behavior, health, and welfare. J. Dairy Sci..

[2] Holden S.A., Butler S.T. (2018). Review: Applications and benefits of sexed semen in dairy and beef herds. Animal.

[3] Probst S., Wasem D., Kobel D., Zehetmeier M., Flury C. (2019). Greenhouse gas emissions from coupled dairy-beef production in Switzerland. Agrar. Schweiz.

[4] Benedek Z., Dublecz K., Koltay I., Fitos G., Várhelyi V., Magyar M., Pirkó B., Baranyai N. (2023). Representative Survey for Evaluating Housing and Manure Handling Technologies of the Hungarian Pig Sector. Animals.

[5] Boneya G. (2021). Sexed semen and major factors affecting its conception rate in dairy cattle. Int. J. Adv. Res. Biol. Sci..

[6] Yotov S., Fasulkov I., Atanasov A., Kistanova E., Sinapov B., Ivanova B., Yarkov D., Zaimova D. (2023). Influence of Ovarian Status and Steroid Hormone Concentration on Day of Timed Artificial Insemination (TAI) on the Reproductive Performance of Dairy Cows Inseminated with Sexed Semen. Animals.

[7] Carslake C., Vázquez-Diosdado J.A., Kaler J. (2020). Machine learning algorithms to classify and quantify multiple behaviours in dairy calves using a sensor: Moving beyond classification in precision livestock. Sensors.

[8] Dimitrov R., Stamatova-Yovcheva K. (2023). MRI Anatomical Investigation of Rabbit Bulbourethral Glands. Animals.

[9] Uwizeye A., de Boer I.J.M., Opio C.I., Schulte R.P.O., Falcucci A., Tempio G., Teillard F., Casu F., Rulli M., Galloway J.N. (2020). Nitrogen emissions along global livestock supply chains. Nat. Food.

[10] Shine P., Murphy M.D. (2022). Over 20 Years of Machine Learning Applications on Dairy Farms: A Comprehensive Mapping Study. Sensors.

[11] Chivarov N., Dimitrov K., Chivarov S. (2023). Algorithm for Autonomous Management of a Poultry Farm by a Cyber-Physical System. Animals.

[12] Stepancheva T., Marinov I., Gergovska Z. (2024). Milking Temperament and Its Association with Test-Day Milk Yield in Bulgarian Murrah Buffaloes. Animals.

[13] Ward S., Pluske J., Plush K., Pluske J., Rikard-Bell C. (2024). Assessing Decision Support Tools for Mitigating Tail Biting in Pork Production: Current Progress and Future Directions. Animals.

[14] He H., Zhu S., Shen L., Chang X., Wang Y., Zeng D., Xiong B., Dai F., Zhao T. (2023). Integrated Analysis of Machine Learning and Deep Learning in Silkworm Pupae (Bombyx mori) Species and Sex Identification. Animals.

[15] Kukar M., Vračar P., Košir D., Pevec D., Bosnić Z.J.C., Agriculture E.I. (2019). AgroDSS: A decision support system for agriculture and farming. Comput. Electron. Agric..

[16] Onuoha C. (2020). Reproductive physiology of male rabbits: A key factor in buck selection for breeding (Paper review). Adv. Reprod. Sci..

[17] Zhang L., Guo W., Lv C., Guo M., Yang M., Fu Q., Liu X. (2024). Advancements in artificial intelligence technology for improving animal welfare: Current applications and research progress. Anim. Res. One Health.

[18] Davis S.E., Lasko T.A., Chen G., Siew E.D., Matheny M.E. (2017). Calibration drift in regression and machine learning models for acute kidney injury. J. Am. Med. Inf. Assoc..

[19] Upadhyay A.K., Khandelwal K. (2019). Artificial intelligence based training learning from application. Dev. Learn. Organ..

[20] Cooke S. (2021). The ethics of touch and the importance of nonhuman relationships in animal agriculture. J. Agric. Environ. Ethics.

[21] Parikh R.B., Helmchen L.A. (2022). Paying for artificial intelligence in medicine. NPJ Digit. Med..

